# Does wearing swimming goggles affect corneal morphology in keratoconic eyes?

**DOI:** 10.5935/0004-2749.20200061

**Published:** 2020

**Authors:** Döndü Melek Ulusoy, Zeynep Duru

**Affiliations:** 1 Department of Ophthalmology, Kayseri Education and Research Hospital, Kayseri, Turkey

**Keywords:** Eye protective devices/adverse effects, Swimming, keratoconus, Corneal pachymetry, Biometry, Dispositivos de proteção dos olhos/efeitos adversos, Natação, Ceratocone, Paquimetria corneana, Biometria

## Abstract

**Purpose:**

A significant transient increase in intraocular pressure in individuals wearing swimming goggles has been demonstrated in previous studies. These findings suggested that wearing swimming goggles could represent a significant risk factor for worsening of corneal parameters in patients with keratoconus who swim regularly. The aim of this study was to investigate corneal parameters in patients with keratoconus after wearing swimming goggles.

**Methods:**

Comprehensive ocular examinations were performed on 74 eyes of 37 patients with keratoconus. Measurements of the corneal front keratometry values (K_flat_, K_steep_, and K_max_), central corneal thickness, corneal apex thickness, thinnest corneal thickness, corneal volume, anterior chamber volume, anterior chamber depth, and iridocorneal angle were performed in outpatient clinics using a Pentacam^®^ Scheimpflug camera (Oculus, Wetzlar, Germany) before the patients wore swimming goggles and after they wore swimming goggles for 1, 10, and 20 min. A p-value of <0.05 was regarded as statistically significant.

**Results:**

The average values before and after wearing swimming goggles for 1, 10, and 20 min were 52.72 ± 5.36, 52.64 ± 5.52, 52.62 ± 5.38, and 52.22 ± 4.86, respectively (p=0.257). The average values before and after wearing swimming goggles for 1, 10, and 20 min were 46.01 ± 3.17, 46.09 ± 3.17, 46.06 ± 3.26, and 46.04 ± 3.17, respectively (p=0.426). The average values before and after wearing swimming goggles for 1, 10, and 20 min were 49.02 ± 3.56, 49.06 ± 3.61, 49.08 ± 3.62, and 49.07 ± 3.61, respectively (p=0.750). No other corneal parameters showed changes after wearing swimming goggles (p>0.05). However, the anterior chamber volume markedly decreased after wearing swimming goggles (p<0.001).

**Conclusions:**

These findings suggested that the short-term use of swimming goggles does not increase the risk of corneal parameter worsening in patients with keratoconus.

## Abstract (pt)

**Objetivo:**

Um aumento transitório significativo da pressão intraocular em indivíduos usando óculos de natação foi demonstrado em estudos anteriores. Esses achados sugerem que o uso de óculos de natação pode representar um fator de risco significativo para o agravamento dos parâmetros corneanos em pacientes com ceratocone que nadam regularmente. O objetivo deste estudo foi investigar os parâmetros corneanos em pacientes com ceratocone após o uso de óculos de natação.

**Métodos:**

Foram realizados exames oftalmológicos completos em 74 olhos de 37 pacientes com ceratocone. As medidas dos valores de ceratometria frontal da córnea (K_flat_, K_steep_ e K_max_), espessura corneana central, espessura apical da córnea, espessura corn eana mais fina, volume da córnea, volume da câmara anterior, profundidade da câmara anterior e ângulo iridocorneano foram realizados em ambulatórios usando câmera Pentacam^®^ Scheimpflug (Oculus, Wetzlar, Alemanha) antes dos pacientes usarem óculos de natação por 1, 10 e 20 min. Um valor p<0,05 foi considerado estatisticamente significativo.

**Resultados:**

Os valores médios de antes e após o uso de óculos de natação por 1, 10 e 20 min foram: 52,72 ± 5,36, 52,64 ± 5,52, 52,62 ± 5,38 e 52,22 ± 4,86, respectivamente (p=0,257). Os valores médios do (antes e após o uso de óculos de natação por 1, 10 e 20 min foram: 46,01 ± 3,17, 46,09 ± 3,17, 46,06 ± 3,26 e 46,04 ± 3,17, respectivamente (p=0,426). Os valores médios de antes e após o uso de óculos de natação por 1, 10 e 20 min foram: 49,02 ± 3,56, 49,06 ± 3,61, 49,08 ± 3,62 e 49,07 ± 3,61, respectivamente (p=0,750). Nenhum outro parâmetro da córnea apresentou alterações após o uso de óculos de natação (p>0,05). No entanto, o volume da câmara anterior diminuiu acentuadamente após o uso de óculos de natação (p<0,001).

**Conclusões:**

Esses achados sugerem que o uso de óculos de natação a curto prazo não aumenta o risco de piora dos parâmetros corneanos em pacientes com ceratocone.

## INTRODUCTION

Swimming is a popular form of physical activity in which swimming goggles are typically worn to reduce or prevent irritation of the eyes, as well as improve underwater vision^(1)^. While wearing swimming goggles can be useful, it can also have adverse effects on the eyes. There have been case reports of supraorbital neuralgia, diplopia, eyelid swelling, migraine, and contact dermatitis associated with the use of swimming goggles^(2-6)^. In addition, previous studies have demonstrated a significant transient increase in intraocular pressure (IOP) in individuals who wear swimming goggles, associated with the excessive negative pressure formed by swimming goggles worn with a very tight seal^(7)^.

The cornea is a viscoelastic tissue that exhibits classic viscoelastic biomechanical properties, such as hysteresis, stress relaxation, elasticity, and creep. It has been shown in recent studies that corneal biomechanical mea surement values are significantly altered in keratoconic eyes, relative to those in normal eyes^(8-11)^. Swimming goggles induce pressure around the periocular area and might affect the corneal parameters in keratoconic eyes. Swimmers who wear goggles are exposed to this pressure, and it is unknown whether this pressure could affect the progression of keratoconus. We presumed that the use of swimming goggles could represent a significant risk factor for worsening of corneal parameters in patients with keratoconus who swim regularly. Therefore, we designed this Zpilot study to objectively assess the impact of wearing swimming goggles on the corneal morphology in patients with keratoconus, using a Pentacam^®^ Scheimpflug camera (Oculus, Wetzlar, Germany).

## METHODS

### Study population

This pilot study was performed at the Department of Ophthalmology, Kayseri Education and Research Hospital. The study protocol followed the tenets of the Declaration of Helsinki and was approved by the local ethics committee. All patients received both oral and written information about the study, and each patient provided written informed consent. A total of 74 eyes from 37 patients with keratoconus were included in this pilot study. Each patient underwent a comprehensive ophthalmic assessment that included best-corrected visual acuity, slit-lamp biomicroscopy, stereoscopic fundus examination, air-puff tonometer, and Pentacam^®^ Scheimpflug camera imaging.

### Pilot design

For the pilot study, 37 patients were recruited from ke ratoconus clinics. Corneal topography measurements were performed using a Pentacam^®^ before swimming goggles were worn. The patients were instructed to wear swimming goggles. After 1 min, they were asked to remove their swimming goggles; topographic measurements were quickly performed, following the removal of the goggles. After a 5 min rest interval, this process was repeated; the goggles were worn for 10 min and 20 min in two respective trials, and measurements were performed after each trial. The same swimming goggles were used for all patients to avoid biasing the results of the study.

### Exclusion criteria

The ocular exclusion criteria for this study included a prior history of significant ocular disease (except for keratoconus), uveitis, ocular trauma, dense media opacities, IOP readings greater than 21 mmHg, or glaucoma. Patients who had undergone any ocular surgery or laser therapy, as well as those with a systemic disease that could affect the anterior segment of the eye, were also excluded from the study.

### Pentacam^®^ Scheimpflug camera measurements

Pentacam^®^ (Oculus) is a rotating Scheimpflug camera system for anterior segment analysis. In this study, three-di mensional anterior chamber analysis modules were used. Measurements were performed in the dark to standardize all measurements for each patient. The patients were asked to focus on a blue fixation light; after placement of the head in the appropriate position, each patient underwent two consecutive measurements to avoid incorrect calculations due to poor imaging quality. For each patient, only one mSeasurement defined as “ok” for examination quality, as determined by the unit, was selected for this study. The central corneal thickness, corneal apex thickness, thinnest corneal thickness, corneal volume, keratometry values (K_flat_, K_steep_ and K_Max_), anterior chamber depth, anterior chamber volume (ACV), and iridocorneal angle were obtained in each Pen tacam^®^ image. All measurements were repeated following the removal of the swimming goggles after wearing them for 1, 10, and 20 min. All of these periods were determined on the basis of the average duration for which swimmers wear swimming goggles.

### Statistical analysis

All statistical tests were performed using SPSS statistical software, version 20.0 (IBM Corp., Armonk, NY, USA). Data were presented as mean ± standard deviation. For each continuous variable, normality was checked by the Kolmogorov-Smirnov test. Data resulting from four examinations (before, after 1 min, after 10 min, and after 20 min of wearing goggles) were analyzed using repeated-measures analysis of variance with Bonferroni’s correction. A p-value of <0.05 was considered statistically significant.

## RESULTS

The mean age of the patients was 25.97 ± 7.7 years (51.3% women, 48.7% men). A comparison of corneal parameters before wearing swimming goggles and after wearing them for 1, 10, and 20 min is presented in table 1. There were no statistically significant differences in any corneal parameters (p>0.05). A comparison of the an terior chamber parameters before wearing swimming goggles and after wearing them for 1, 10, and 20 min is shown in table 2. The ACV was significantly reduced after wearing swimming goggles (p<0.001), whereas other corneal anterior chamber parameters did not change (p>0.05).

**Table 1:** Mean changes in corneal parameters before and after wearing goggles

	Before wearing	1 min	10 min	20 min	p-value^a^
CCT (µm)					
*Mean ± SD*	472.7 ± 36.7	473.1 ± 35.6	473.2 ± 36.9	474.2 ± 36.0	0.180
CAT (µm)					
*Mean ± SD* TCT (µm)	466.1 ± 40.1	466.6 ± 38.9	466.7 ± 40.4	467.8 ± 39.3	0.119
*Mean ± SD*	457.4 ± 40.3	457.2 ± 38.4	457.9 ± 40.8	459.0 ± 39.2	0.084
CV (mm^3^)					
*Mean ± SD*	57.7 ± 3.8	56.5 ± 3.9	56.6 ± 3.9	56.8 ± 3.8	0.157
*K*_flat_ (D)					
*Mean ± SD*	46.01 ± 3.17	46.09 ± 3.17	46.06 ± 3.26	46.04 ± 3.17	0.426
*K*steep (D)					
*Mean ± SD*	49.02 ± 3.56	49.06 ± 3.61	49.08 ± 3.62	49.07 ± 3.61	0.750
K_max_ (D)					
*Mean ± SD*	52.72 ± 5.36	52.64 ± 5.52	52.62 ± 5.38	52.22 ± 4.86	0.493

SD= standard deviation; D= diopter; CCT= central corneal thickness; CAT= corneal apex thickness; TCT: thinnest corneal thickness; CV= corneal volume; max= maximum.

a = Repeated-measures analysis of variance (ANOVA) with Bonferroni’s correction (p<0.05).

**Table 2:** Mean changes in anterior chamber parameters before and after wearing goggles

	Before wearing	1 min	10 min	20 min	p-value^a^
ACV (mm^3^)					
*Mean ± SD*	206.8 ± 34.0^b^	203.8 ± 32.9^C^	202.5 ± 32.2^C^	202.5 ± 31.8^C^	<0.001
ACD (mm)					
*Mean ± SD*	3.73 ± 0.39	3.72 ± 0.38	3.71 ± 0.38	3.71 ± 0.39	0.220
ICA(°)					
*Mean ± SD*	39.22 ± 4.83	39.37 ± 5.05	39.25 ± 5.17	39.30 ± 5.35	0.995

SD= standard deviation; ACV= anterior chamber volume; ACD= anterior chamber depth; ICA= iridocorneal angle.

a = Repeated-measures ANOVA with Bonferroni’s correction (p<0.05).

b,c = Different letters in the same column indicate statistical significance.

## DISCUSSION

Various studies have documented the effects of using swimming goggles on IOP. In a study by Morgan et al., the researchers reported an increase in IOP due to the use of swimming goggles, and they concluded that swimming goggles with smaller frames had a greater effect on IOP compared to swimming goggles with larger frames^(7)^. In another study, Paula et al. reported that swimming goggles significantly increased IOP after 2 min of use; after removing the swimming goggles, the IOP decrea sed significantly^(12)^. Furthermore, Ma et al. reported a small but significant IOP elevation immediately after the swimming goggles were affixed to the face. This elevated IOP was maintained while the goggles were worn, and then it returned to the normal level as soon as they were removed^(13)^. Unlike the studies of IOP, the effects of wearing swimming goggles on corneal parameters are not well addressed in the ophthalmic liSterature. Our findings in the present study suggested that the use of swimming goggles could represent a significant risk factor for worsening of corneal parameters in patients with keratoconus who swim regularly.

Keratoconus is a progressive disorder in which the cornea assumes a conical shape as a result of noninflammatory thinning and protrusion^(14,15)^. It has been reported in recent investigations that corneal biomechanical values are significantly altered in keratoconic eyes, relative to normal eyes^(8-11)^. Furthermore, studies have shown that an elastic material can regain its original form in a completely reversible displacement along the stress-strain pathway when the imposed stress is removed^(16)^.

In response, we hypothesized that the corneal morphology could be affected by wearing swimming goggles. A possible mechanism for the worsening of corneal parameters is that the headband tension of the swimming goggles is transmitted into the orbit through the rubber seal, thereby increasing the orbital tissue pressure, compressing the globe, and causing alterations in the corneal morphology. An alternative possible mechanism is that the vacuum pressure inside the chamber formed by the goggles around each eye might also affect the corneal morphology.

This preliminary study was performed on patients with keratoconus and simulated only the short-term effects of using swimming goggles. Our study showed no alterations in corneal parameters after swimming goggles were worn. Corneal keratometric values, corneal thickness values, and anterior chamber parameters showed no significant changes. In contrast, the ACV decreased markedly after swimming goggles were worn. This suggests that using swimming goggles affects anterior chamber parameters but does not affect corneal parameters. We speculate that the negative pressure created by swimming goggles might move the iris-lens diaphragm anteriorly, in relation to the temporary increase in the IOP. In our opinion, the minimal change in ACV that we observed might be dependent on this movement of the iris-lens diaphragm. Another possible reason might be that the elastic compression transmitted to the eyes by the swimming goggles might temporarily increase the aqueous humor outflow, leading to the reduction of the ACV. Although it was statistically significant, we presume that this minimal reduction in the ACV was not clinically significant.

Our study had some limitations. First, we did not assess the effects of potential pressure changes underwater. Second, we were unable to perform topographic measurements while the patients were wearing the swimming goggles. However, we performed topographic measurements immediately after the removal of the swimming goggles. Third, in this study, we used one common type of swimming goggles; different goggle designs with differences in pressure around the periorbital tissue could cause distinct effects on the corneal morphology.

In summary, based on the findings of our study, the short-term use of swimming goggles does not affect keratometric and corneal thickness values in patients with keratoconus. Thus, using swimming goggles may not cause progression in keratoconic eyes. However, additional studies are needed to assess the long-term relationship between the use of swimming goggles and progression of keratoconus.
